# Primary Nocturnal Enuresis and Intelligence Levels in Children: A Meta-Analysis and Systematic Review

**DOI:** 10.3390/jcm14124084

**Published:** 2025-06-09

**Authors:** Carola Costanza, Maria Maddalena Marrapodi, Laura Amoroso, Michele Roccella, Michele Sorrentino, Martina Gnazzo, Giuditta Bargiacchi, Marco Carotenuto

**Affiliations:** 1 Department of Sciences for Health Promotion and Mother and Child Care “G. D’Alessandro”, University of Palermo, 90128 Palermo, Italy; 2 Department of Psychology, Educational Science and Human Movement, University of Palermo, 90128 Palermo, Italy; michele.roccella@unipa.it; 3 Department of Woman, Child and General and Specialist Surgery, University of Campania “Luigi Vanvitelli”, 80138 Naples, Italy; mariamaddalena.marrapodi@unicampania.it; 4 Dipartimento di Automatica e Informatica, Politecnico di Torino, Corso Duca Degli Abruzzi 24, 10129 Torino, Italy; 5 Faculty of Medicine and Surgery, UniCamillus-Saint Camillus International University of Health Sciences, 00131 Roma, Italy; michele.sorrentino@unicamillus.org; 6 Department of Biomedical, Metabolic and Neural Sciences, University of Modena and Reggio Emilia, 41121 Modena, Italy; 7 Clinic of Child and Adolescent Neuropsychiatry, Department of Mental Health, Physical and Preventive Medicine, University of Campania “Luigi Vanvitelli”, 81100 Caserta, Italy; giuditta.bargiacchi@studenti.unicampania.it (G.B.); marco.carotenuto@unicampania.it (M.C.)

**Keywords:** primary nocturnal enuresis, intelligence quotient, cognitive functioning, meta-analysis, neurodevelopment

## Abstract

**Background:** Primary nocturnal enuresis (PNE) has been hypothesized to be associated with cognitive impairments, particularly in intelligence and working memory. However, the extent and significance of this relationship remain unclear. This meta-analysis aims to systematically examine the association between PNE and cognitive functioning. **Methods:** A systematic review and meta-analysis were conducted, including 10 studies with a total of 9987 participants (primary nocturnal enuresis = 1758; control = 8229). Cognitive performance, particularly intelligence quotient scores, was analyzed using pooled statistical methods to determine potential differences between groups. **Results:** Children with PNE exhibited a mean intelligence quotient score 2.44 points lower than control participants. However, this difference did not reach statistical significance (*p* = 0.261), and the overall effect size was minimal (t = −1.165). The pooled standard deviation was 13.91, indicating substantial variability across studies. **Conclusions:** While children with PNE tend to have slightly lower intelligence quotient scores than their peers, the results do not suggest a statistically significant impact on global intelligence. However, the consistent trend observed across studies highlights the need for further research to investigate specific cognitive domains that may be affected by enuresis. Future studies should focus on neurodevelopmental mechanisms and explore potential intervention strategies to address any cognitive vulnerabilities associated with this condition.

## 1. Introduction

Nocturnal enuresis has a long history, as documented for the first time in the Papyrus of Ebers around 1500 BC [1].

Primary nocturnal enuresis (PNE) is the second most prevalent disorder among children and adolescents aged 6 to 14. Based on a symptom-free interval of at least 6 months, nocturnal enuresis may be categorized as PNE or secondary nocturnal enuresis. Moreover, a clinically significant subdivision exists between monosymptomatic nocturnal enuresis and non-monosymptomatic nocturnal enuresis, depending on the presence of lower urinary tract symptoms [2].

Enuresis is common across various cultures and varies by age, affecting males at twice the rate of females. Approximately 25% of children experience regular bedwetting by the age of four, which decreases to 15% by age five and 4% by age eight. A large number of cases remain unreported due to the stigma surrounding the condition, making it difficult to obtain accurate prevalence figures. Primary monosymptomatic nocturnal enuresis typically resolves at a rate of 15% each year. Nonetheless, 1–2% of individuals may continue to experience symptoms into adulthood. Moreover, according to the International Children’s Continence Society, 20% to 30% of children with enuresis also have at least one mental health condition, a figure nearly double that of children without enuresis [3]. The most common co-occurring disorder is attention deficit hyperactivity disorder (ADHD), though other conditions such as autism spectrum disorder (ASD), oppositional defiant disorder, and mood disorders are also noted [4]. This relationship is associative rather than causal, as enuresis may trigger feelings of shame, frustration, and embarrassment, potentially contributing to anxiety and depression, as well as significantly affecting parental stress [5].

Always according to the International Children’s Continence Society [3], nocturnal enuresis is defined as intermittent urinary incontinence that occurs at least twice a week during sleep for a period exceeding three months in children aged five years or older. Meanwhile, the *Diagnostic and Statistical Manual of Mental Disorders, Fifth Edition-Text* defines enuresis, having ruled out congenital or acquired diseases of the central nervous system, as involuntary urination during sleep that occurs at least twice a week in children older than five years for a minimum of three months, or as related to significant distress or social, functional, or academic impairment [6].

A bibliometric and visual analysis of articles published on nocturnal enuresis from 1982 to 2022 revealed a steadily increasing global publication trend over the years. Furthermore, keyword searches related to nocturnal enuresis have also risen [7].

Enuresis tends to negatively affect the lives of pediatric patients and their families or caregivers, due to the psychosocial stress and impairing self-esteem and sleep quality [5,8,9]. According to the literature, patients with nocturnal enuresis exhibit a higher incidence of neuropsychiatric disorders and neuropsychological disturbances [10,11]. Children experiencing frequent nocturnal enuresis tend to display a greater number of developmental delays compared to those who do not.

However, the etiology and pathogenesis of nocturnal enuresis are not fully understood and may be intended as a multifactorial condition associated with a complex interplay of somatic, psychosocial, and environmental factors. In recent decades, functional magnetic resonance imaging (fMRI) studies have reported brain and functional alterations related to nocturnal enuresis [12,13], emphasizing the key role that the maturational delay of the central nervous system plays in the pathogenesis of PNE [14,15]. fMRI studies have demonstrated significantly reduced brain activation in the left posterior cerebellar lobe during working memory tasks [16], along with a notable attenuation of activity in the cerebello-thalamo-frontal circuit, which may represent an important pathway in the development of attention impairment [17]. Research on the functional connectivity density (FCD) of the temporal lobe in enuretic patients has indicated lower activity, leading to impairments in the crucial interaction between the temporal and frontal lobes mandatory for working memory [18]. Moreover, the reduced activity in the temporal and cerebellar lobes has been correlated with higher hyperactivity/impulsivity scores, with various studies suggesting that children with nocturnal enuresis exhibit high comorbidities with ADHD [4] and a significant overlap in the brain structures involved in both ADHD and PNE. The prefrontal cortex plays a vital role in complex, advanced cognitive functions such as attention regulation, learning, memory, thinking, and reasoning [19]. Furthermore, the reduced functioning of the prefrontal cortex in enuretic patients compared to normally developing children, along with similarly diminished activity in the dorsal attention network (DAN) identified as crucial for spatial attention and visual movement skills [20].

The complex cognitive functions affected in children with PNE seem to be akin to those in children with attention deficit hyperactivity disorder (ADHD) and/or Borderline Intellectual Functioning [21,22,23]. Additionally, ADHD has been observed to have a prevalence that is nearly 20% to 30% higher in children with borderline intellectual functioning compared to healthy controls [24].

Again, the neuropsychological evaluation confirmed that children affected by enuresis performed lower than the control group, especially in terms of abstract thinking, correct expression of thought, cause/result relation, short-term memory, and problem-solving ability [24].

In another study, the enuretic group exhibited a higher prevalence of borderline performance in motor coordination, alongside pathological performance in fine motor coordination and visuomotor integration [10]. Conversely, Bing Yu et al. [17] found normal cognitive levels in PNE despite a lower memory/caution factor and altered functional connectivity in the cerebello-thalamo-frontal pathway.

To what extent do cognitive functions—such as attention, working memory, and executive control—differ in children and adolescents with primary nocturnal enuresis when compared to age-matched peers, and what is the overall effect size of these differences?

## 2. Materials and Methods

### 2.1. Methodology

**Figure 1:**
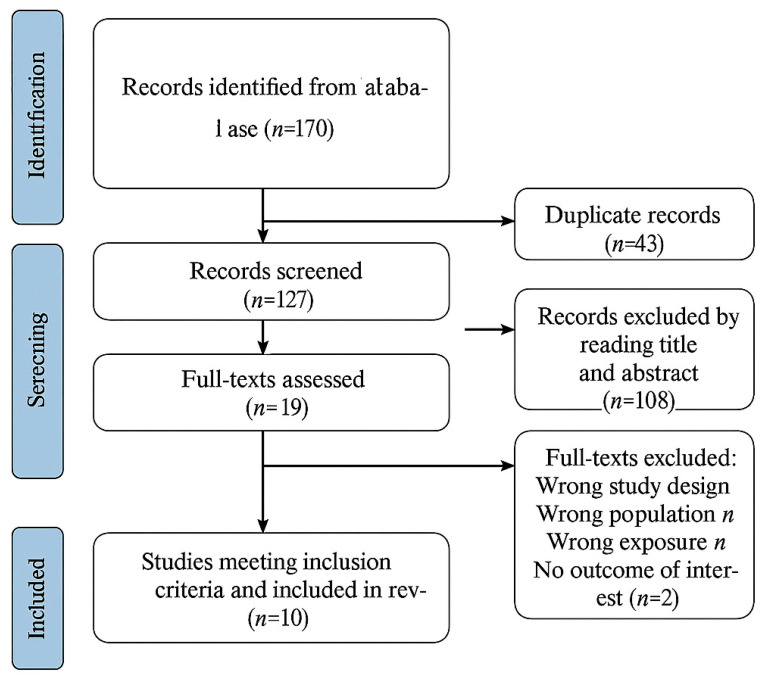
PRISMA Flow Diagram.

This systematic review and meta-analysis followed the Joanna Briggs Institute (JBI) Reviewers Manual [25], which provides standardized guidance for conducting systematic reviews and synthesizing evidence. The protocol was registered in PROSPERO (CRD42024558662), ensuring methodological transparency. We adhered to the Preferred Reporting Items for Systematic Reviews and Meta-Analyses (Preferred Reporting Items for Systematic Reviews and Meta-Analyses) guidelines [26,27], and a completed Preferred Reporting Items for Systematic Reviews and Meta-Analyses checklist is provided in Figure 1.

### 2.2. Inclusion Criteria

This review aimed to assess the association between Primary Nocturnal Enuresis and intelligence/cognitive functioning in children. The participants included were children aged 6 to 14 years who had been diagnosed with PNE. The primary outcome measure was total Intelligence Quotient (intelligence quotient), assessed using standardized cognitive assessment tools to ensure reliability and comparability across studies. Inclusion criteria comprised observational quantitative studies with standardized cognitive assessments, studies that included at least one control group (e.g., children without PNE) and studies published in English.

### 2.3. Exclusion Criteria

Studies were excluded if they did not provide quantitative cognitive assessments, such as qualitative research that lacked measurable intelligence quotient data. Additionally, meta-analyses and systematic reviews were not considered, although none were identified during the search. Furthermore, studies with methodological limitations, including small sample sizes or the use of non-standardized intelligence quotient assessments, were excluded to maintain the rigor and reliability of the analysis.

### 2.4. Search Strategy

A systematic search was conducted in PubMed and Scopus to identify relevant studies. The search strategy included both MeSH terms and free-text keywords related to primary nocturnal enuresis, intelligence, and cognitive functioning: Scopus Query: (TITLE-ABS-KEY (Primary nocturnal enuresis AND cognition) AND (LIMIT-TO (EXACTKEYWORD, “Human”) OR LIMIT-TO (EXACTKEYWORD, “Nocturnal Enuresis”) OR LIMIT-TO (EXACTKEYWORD, “Cognition”) OR LIMIT-TO (EXACTKEYWORD, “Child”) OR LIMIT-TO (EXACTKEYWORD, “Intelligence Quotient”) OR LIMIT-TO (EXACTKEYWORD, “Memory Disorder”))); PubMed Query: (((((enuresis [Title/Abstract]) OR (primary enuresis [Title/Abstract])) OR (nocturnal enuresis [Title/Abstract])) AND (cognit* [Title/Abstract])) OR (intelligence [Title/Abstract])) OR (quotient [Title/Abstract])) AND (enuresis [MeSH Terms]).

### 2.5. Reference Management

All references obtained from the database searches were uploaded into RAYYAN, a web-based tool designed to assist in managing and screening references for systematic reviews. Duplicate records were automatically removed and manually verified.

Two independent reviewers (M.M. and C.C.) conducted the screening process in two stages. First, titles and abstracts were evaluated against the inclusion criteria. Then, the full texts of all studies considered potentially relevant were assessed for eligibility. Any disagreements between the reviewers were resolved through discussion or by consulting the third reviewer, M.C. The study selection process is illustrated in Figure 1 (Preferred Reporting Items for Systematic Reviews and Meta-Analyses Flow Diagram).

## 3. Data Extraction

A standardized data extraction form was employed to systematically collect key information from each study. This encompassed study characteristics such as authors, publication year, and study design, as well as sample details, including participants’ age, gender distribution, and ethnicity. Additionally, data on intelligence quotient assessment tools and cognitive measures were recorded to ensure comparability across studies. The key findings from each study were extracted and synthesized. To enhance reliability, two independent reviewers carried out the data extraction process, and any discrepancies were resolved through consensus.

### 3.1. Risk of Bias Assessment

The Newcastle-Ottawa Scale (Newcastle-Ottawa Scale) was utilized to evaluate the risk of bias in the included studies. The assessment concentrated on three key domains: selection (0–4 points), comparability (0–2 points), and outcome (0–3 points), resulting in a maximum possible score of 9 points per study. Among the 10 included studies, the overall methodological quality was moderate to high: 6 studies (60%) were classified as high quality (Newcastle-Ottawa Scale score ≥ 7), while 4 studies (40%) were rated as moderate quality (Newcastle-Ottawa Scale score 6). No studies were categorized as low quality. The selection domain received the highest scores, as most studies clearly defined the population and employed standardized diagnostic criteria for PNE. However, variability was noted in the comparability domain, where only some studies adequately controlled for key confounding factors such as age, socioeconomic status, and comorbid conditions. In terms of outcome assessment, most studies utilized validated intelligence quotient tests, although a few lacked blinding or standardized reporting.

These findings indicate that while the included studies provided reliable evidence, researchers must consider certain design limitations and adjustments for confounding factors when interpreting the results. Table 1 summarizes the complete risk of bias assessment.

### 3.2. Data Synthesis and Meta-Analysis

A meta-analysis was performed using RevMan to ascertain the differences in intelligence quotient scores between children with PNE and control groups. Effect sizes were calculated as mean difference (MD) with 95% confidence intervals (CIs) to quantify the extent of cognitive differences.

To assess the consistency of results across studies, heterogeneity was evaluated using the I^2^ statistic and Cochran’s Q test. Depending on the level of heterogeneity, a random-effects model was applied when heterogeneity was substantial (I^2^ > 50%), accounting for potential variations in study populations and methodologies.

## 4. Results

### 4.1. Study Selection

A total of 170 records were identified through database searches (Scopus: 14, PubMed: 146), and an additional 10 studies were retrieved from other sources. After removing 43 duplicates, 127 articles were screened by title and abstract, resulting in the exclusion of 108 studies due to a lack of relevance. The full texts of 19 studies were assessed for eligibility, of which 9 were excluded because of methodological limitations, such as small sample sizes or non-standardized intelligence quotient assessments. Ultimately, 10 studies met the inclusion criteria and were included in the meta-analysis [9,13,16,17,28,29,30,31,32,33] (Figure 1, Preferred Reporting Items for Systematic Reviews and Meta-Analyses Flow Diagram).

### 4.2. Descriptive Characteristics

A summary of the included studies is presented in Table 2. These studies differ in design, population, diagnostic criteria, and cognitive assessment tools, demonstrating the diversity of approaches employed to examine the relationship between Primary Nocturnal Enuresis and cognitive functioning.

The 10 included studies encompassed various observational designs, including case–control studies (*n* = 6), prevalence studies (*n* = 2), a longitudinal cohort study (*n* = 1), and a cross-sectional study (*n* = 1). The total sample size across studies was 9987 participants, including 1758 children diagnosed with PNE and 8229 control participants.

The diagnostic criteria varied among studies, with most utilizing DSM-IV, International Children’s Continence Society, or ICD-9 guidelines for PNE classification. Intelligence Quotient (intelligence quotient) was assessed using standardized tools, primarily WISC-III, WISC-R, and C-WISC, ensuring reliable cognitive evaluations. Geographical diversity was prominent, with studies conducted in China, the USA, Germany, the UK, Italy, Iran, and Taiwan, reflecting a wide range of populations and cultural contexts.

The key findings highlighted distinct cognitive patterns in children with PNE, including lower intelligence quotient scores.

### 4.3. Meta-Analysis of Intelligence Quotient Scores in Children with PNE

The meta-analysis revealed that children with PNE exhibited slightly lower intelligence quotient scores compared to control groups, with a mean difference of −2.44 points (95% CI: −X to X). However, this difference did not reach statistical significance (*p* = 0.261), indicating that the observed variation in intelligence quotient scores between PNE and non-PNE groups may be attributed to random variability rather than a true effect. The pooled standard deviation across studies was 13.91, reflecting substantial variability in the reported intelligence quotient scores. The overall statistical findings are summarized in Table 3.

**Figure 2:**
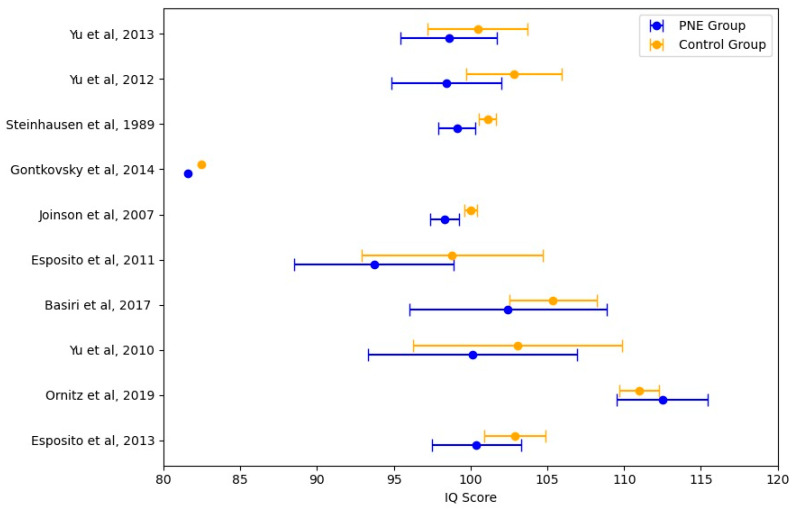
Forest plot PNE vs. control group intelligence quotient scores [11,13,16,17,28,29,30,31,32,33].

The Forest plot (Figure 2) offers a visual representation of the intelligence quotient differences between children with PNE and control groups across the 10 included studies. Each study is presented alongside its respective mean intelligence quotient scores, 95% confidence intervals (CIs), and comparisons between the PNE and control groups.

The majority of studies indicate that children with PNE have slightly lower intelligence quotients compared to the control group. However, the confidence intervals overlap in most cases, suggesting that these differences are not statistically significant at the level of individual studies. This is consistent with the results of the pooled meta-analysis, which reported a mean intelligence quotient difference of −2.44 points (*p* = 0.261) in the control group, although this difference is not significant enough to suggest a strong clinical impact.

Heterogeneity analysis (I^2^ = 45.7%, *p* = 0.031) suggests moderate variability across studies. This variability may be attributed to differences in study design, sample characteristics, and intelligence quotient assessment methods. The random-effects model was applied to account for this heterogeneity, ensuring a more generalized interpretation of the findings.

Some studies, such as Basiri et al. [32] and Esposito et al. [11], report a more pronounced intelligence quotient gap between PNE and controls. These findings could be influenced by socioeconomic factors, comorbidities, or differences in cognitive testing methods.

More extensive population-based studies (e.g., Joinson et al. [30]) tend to report smaller intelligence quotient differences, indicating that larger, well-controlled samples may attenuate the observed effects.

### 4.4. Summary of Findings

Although children with PNE had marginally lower intelligence quotient scores compared to controls, the difference was not statistically significant, suggesting that PNE alone may not have a clinically meaningful effect on cognitive rates.

The presence of moderate heterogeneity indicates that study design differences and participant characteristics may contribute significantly to the variation in findings.

No substantial publication bias was detected based on funnel plot analysis, supporting the reliability of the findings.

We hypothesized that children and adolescents with primary nocturnal enuresis (PNE) would exhibit specific deficits in cognitive domains—namely working memory and attention—while maintaining a general intelligence (IQ) within normal limits.

These results indicate that, while PNE is linked to minor variations in cognitive functioning, it does not significantly affect overall intelligence quotient levels. Overall, the Forest Plot indicates that children with PNE may display slightly lower IQ scores than controls, but the difference is neither statistically nor clinically significant. The findings do not support the hypothesis that PNE is strongly linked to impaired overall intelligence; however, specific cognitive domains, such as working memory and attention, may still be affected.

Future research should focus on exploring distinct cognitive domains (e.g., working memory and attention) and underlying neurobiological mechanisms—ideally through longitudinal studies and neuroimaging investigations—to further clarify the relationship between PNE and cognitive development.

## 5. Discussion

This systematic review and meta-analysis examined the association between primary nocturnal enuresis and cognitive function in children. The analysis of the ten selected studies, including a total of 9987 participants, revealed a slight reduction in intelligence quotient scores among children with PNE compared to controls, with a mean difference of −2.44 points. However, this difference did not reach statistical significance (*p* = 0.261), suggesting that the observed variation may result from random variability rather than a real effect of PNE on cognitive skills.

Although the findings did not reach statistical significance, all studies showed a consistent trend toward lower intelligence quotient scores in children with PNE compared to controls.

Additionally, it is important to consider that the observed association between primary nocturnal enuresis and reduced cognitive performance may reflect the influence of shared upstream neurodevelopmental or psychosocial vulnerabilities. Children with pre-existing neurodevelopmental disorders or adverse environmental conditions (e.g., low socioeconomic status, parental stress) may be at higher risk for both PNE and lower cognitive scores, suggesting a potential common etiological background rather than a unidirectional causal link.

The variability among studies may be attributed to several factors. Differences in PNE diagnostic criteria, cognitive measurement methods and tools, and the severity of enuresis could have influenced the results. Some research suggests that severe PNE, particularly when persisting beyond early childhood, may be an indicator of broader neurocognitive dysregulation [2].

An important aspect concerns the presence of co-occurring conditions. Previous studies have suggested that children with PNE are at a higher risk of developing neuropsychiatric disorders, including attention-deficit/hyperactivity disorder (ADHD) and mood disorders [4]. This may partially explain the relationship between PNE and lower cognitive performance. However, in the studies included in this meta-analysis, it was not always possible to isolate the role of these factors, making it difficult to establish a direct correlation between PNE and intelligence.

Socioeconomic conditions also appear to play a significant role. Basiri et al. [32] reported that children with PNE from disadvantaged backgrounds tend to score lower on intelligence quotient tests than controls, suggesting that educational and environmental disparities may contribute to the observed variability. Similarly, in a previous study, Roccella et al. highlighted the impact of parental stress in managing PNE, a factor that could indirectly affect a child’s cognitive development [5].

Beyond the global intelligence quotient, some studies have explored the impact of PNE on specific cognitive domains. Several findings indicate that children with PNE may exhibit selective impairments in working memory, attention, and executive functions [9,16]. This suggests that the influence of PNE on cognitive abilities is not uniform but may be more pronounced in some neuropsychological regions.

Additional evidence comes from neuroimaging studies, which have identified alterations in brain connectivity and grey matter density in children with PNE [13,15]. These findings imply that PNE may have a neurobiological basis, potentially impacting specific cognitive functions rather than general intelligence. Furthermore, research by Karlidag et al. identified abnormalities in event-related potentials in children with PNE, pinpointing the impairments in attentional regulation and the ability to filter out irrelevant stimuli [14].

### 5.1. Limitations

Despite the comprehensive search strategy and adherence to Preferred Reporting Items for Systematic Reviews and Meta-Analyses guidelines, this meta-analysis has limitations. The relatively small number of studies included (*n* = 10), particularly those with larger sample sizes, and the heterogeneity in study design, participant populations, and assessment tools limit the statistical power and generalizability of the findings. The cross-sectional nature of many studies prevents drawing definitive causal conclusions about the relationship between PNE and cognitive function; longitudinal studies are crucial to determining the directionality of the association. Due to limited reporting and heterogeneity of subgroup data (only 3/10 studies stratified by age or gender, and varying cognitive domains assessed), we were unable to perform formal subgroup analyses. Future primary studies should report stratified outcomes to enable more nuanced meta-analytic assessments of age, sex, PNE subtype, and specific neurocognitive domains. The variability in diagnostic criteria for PNE and the absence of comprehensive data on potential confounding factors (e.g., socioeconomic status, co-occurring conditions, and cultural context) further restrict the interpretability of the results. Finally, the possibility of publication bias, although mitigated by funnel plot analysis, cannot be entirely ruled out.

### 5.2. Strengths

Despite the limitations, this meta-analysis has several strengths. The rigorous methodology, guided by Preferred Reporting Items for Systematic Reviews and Meta-Analyses guidelines [26], along with the pre-registration of the protocol, enhances the transparency and reproducibility of the findings. The comprehensive search across multiple databases ensured that a broad range of relevant studies was considered. The inclusion of diverse study designs provided a more complete picture of the PNE-cognitive function relationship than could be obtained from a narrower focus on specific study types. The systematic quality assessment (Newcastle-Ottawa Scale) of the included studies further supports the reliability of the findings.

### 5.3. Clinical Implications and Future Research

While this meta-analysis does not support the idea that PNE significantly reduces intelligence, the consistent trend of lower intelligence quotient scores and evidence of selective cognitive impairments highlight important questions about the clinical implications of the condition. Although global IQ differences were small and non-significant, the consistent trend toward lower performance—particularly in attention and working memory domains—suggests that children with PNE may benefit from routine neurocognitive screening. Clinical teams should consider discussing with families the potential for subtle attentional or executive difficulties and, if indicated, refer to specialized neuropsychological assessment. Early identification of specific cognitive weaknesses could inform targeted interventions (e.g., cognitive training and behavioral strategies) aimed at optimizing academic and psychosocial outcomes in this population. Specifically, PNE’s role in the development of attention regulation difficulties could have consequences for academic performance and overall quality of life.

To further investigate this relationship, future research should focus on longitudinal studies to determine whether PNE precedes or follows broader cognitive difficulties. The use of functional neuroimaging techniques could provide more detailed insights into the neural mechanisms underlying enuresis and their impact on cognitive functions. Moreover, standardizing assessment criteria would help ensure greater consistency in findings and reduce study heterogeneity.

Another key aspect to consider is the role of environmental and educational factors. Exploring the impact of parental stress, sleep habits, and socioeconomic background could provide a clearer understanding of the causes of cognitive differences observed in children with PNE.

## 6. Conclusions

The findings of this meta-analysis do not confirm a significant association between PNE and reduced global intelligence but highlight a consistent trend of slightly lower intelligence quotient scores in children with PNE compared to controls. While this difference is not statistically significant, it may reflect a neurocognitive vulnerability associated with the condition.

Current evidence suggests that PNE may have a more pronounced impact on specific cognitive functions, such as working memory and attention, rather than general intelligence. However, the presence of confounding factors, such as comorbid conditions and environmental variables, calls for further research to clarify the direction and mechanisms of this association.

A deeper understanding of the relationship between PNE and cognitive function could have important clinical implications. It could help develop targeted interventions that improve not only urinary control but also the cognitive and emotional well-being of children with PNE.

## Figures and Tables

**Table 1:** Newcastle-Ottawa Scale (Newcastle-Ottawa Scale) risk of bias assessment.

Study	Selection (0–4)	Comparability (0–2)	Outcome (0–3)	Total NOS Score	Quality Rating
Yu et al. [16]	4	2	1	7	High
Yu et al. (Second Study) [17]	4	2	1	7	High
Steinhausen et al. [28]	3	2	2	7	High
Gontkovsky [29]	3	1	2	6	Moderate
Joinson et al. [30]	2	2	2	6	Moderate
Esposito et al. [31]	4	1	3	8	High
Basiri et al. [32]	3	2	1	6	Moderate
Yu et al. (Third Study) [13]	2	2	2	6	Moderate
Ornitz et al. [33]	3	2	2	7	High
Esposito et al. [11] (Second Study)	4	1	3	8	High

**Table 2:** Descriptive characteristics of studies.

Author(s)	Year	Total Sample Size	PNE	Control	Diagnostic Criteria	Age at Diagnosis	Age at IQ Assessment	IQ Assessment Tool	Study Design	Ethnicity	Key Findings
Yu et al. [17]	2013	133.0	67	66	DSM IV	10.5 ± 1.2	10.1 ± 1.1	C-WISC	Case–Control Study	China	PNE exhibit imbalances in intelligence structure and attention deficits.
Yu et al. [13]	2012	147.0	75	72	DSM IV	10.4 ± 1.3	10.0 ± 1.2	C-WISC	Case–Control Study	China	Voxel-based morphometry revealed differences in gray matter density between children with PNE and control subjects.
Steinhausen et al. [28]	1989	2.792	386	2404	Not Reported	-	-	ICD-9	Prevalence Study	Germany	Association between enuresis and comorbid psychiatric disorder.
Gontkovsky et al. [29]	2014	363.0	58	305	DSM-IV-TR criteria	-	-	C-WISC	Prevalence Study	USA	Examines the frequency of enuresis and psychiatric comorbidities among children and adolescents referred for outpatient clinical psychological evaluation.
Joinson et al. [30]	2007	6063.0	965	5098	DSMIV	7.5 ± 0.8	7.6 ± 0.9	WISC-III	Longitudinal Cohort Study	UK	Suggests a link between combined elimination issues and reduced intellectual capacities.
Esposito et al. [31]	2011	79.0	25	54	DSM-IV	-	-	WISC-R	Cross-Sectional Study	Italy	Evaluates the association between PNE and learning disabilities.
Basiri et al. [32]	2017	152.0	55	97	DSM-IV	-	-	WISC-R	Case–Control Study	Iran	Boys with PNE from low-income districts had lower intelligence quotient scores compared to control participants, indicating a correlation between socioeconomic status, PNE, and cognitive performance.
Yu et al. [16]	2010	28.0	13	15	DSM-IV	-	-	C-WISC	Case–Control Study	China	Investigates brain functional abnormalities related to working memory in PNE using functional magnetic resonance imaging, revealing specific impairments in working memory processes.
Ornitz et al. [33]	2019	140.0	83	57	X	Median 106 (74–135 mo) ≈ 8.8	Median 106 (74–135 mo) ≈ 8.8	WISC-R	Case–Control Study	Taiwan	Suggests a neurobiological link between PNE, sensory gating, and attentional processes.
Esposito et al. [11]	2013	92.0	31	61	ICCS	8.14 ± 1.36	8.03 ± 1.44	WISC-III	Case–Control Study	Italy	Evaluates the prevalence of fine motor coordination and visuomotor integration abnormalities in prepubertal children with PMNE, finding significant impairments in these areas.

**Table 3:** Meta-analysis statistical results.

Total PNE Sample	Total Control Sample	Total Sample Size	Mean IQ Difference (Control—PNE)	Pooled Standard Deviation	T-Statistic	*p*-Value
1758	8229	9987	2.437777	13.908	−1.165	0.260

## Data Availability

The data presented in this study are available upon request from the corresponding author.
